# Spontaneous hyaline cartilage regeneration can be induced in an osteochondral defect created in the femoral condyle using a novel double-network hydrogel

**DOI:** 10.1186/1471-2474-12-49

**Published:** 2011-02-22

**Authors:** Masashi Yokota, Kazunori Yasuda, Nobuto Kitamura, Kazunobu Arakaki, Shin Onodera, Takayuki Kurokawa, Jian-Ping Gong

**Affiliations:** 1 Department of Sports Medicine and Joint Surgery, Hokkaido University School of Medicine, Sapporo, Japan; 2 Department of Biological Sciences, Graduate School of Science, Hokkaido University, Sapporo, Japan

## Abstract

**Background:**

Functional repair of articular osteochondral defects remains a major challenge not only in the field of knee surgery but also in tissue regeneration medicine. The purpose is to clarify whether the spontaneous hyaline cartilage regeneration can be induced in a large osteochondral defect created in the femoral condyle by means of implanting a novel double-network (DN) gel at the bottom of the defect.

**Methods:**

Twenty-five mature rabbits were used in this study. In the bilateral knees of each animal, we created an osteochondral defect having a diameter of 2.4-mm in the medial condyle. Then, in 21 rabbits, we implanted a DN gel plug into a right knee defect so that a vacant space of 1.5-mm depth (in Group I), 2.5-mm depth (in Group II), or 3.5-mm depth (in Group III) was left. In the left knee, we did not apply any treatment to the defect to obtain the control data. All the rabbits were sacrificed at 4 weeks, and the gross and histological evaluations were performed. The remaining 4 rabbits underwent the same treatment as used in Group II, and real-time PCR analysis was performed at 4 weeks.

**Results:**

The defect in Group II was filled with a sufficient volume of the hyaline cartilage tissue rich in proteoglycan and type-2 collagen. The Wayne's gross appearance and histology scores showed that Group II was significantly greater than Group I, III, and Control (p < 0.012). The relative expression level of type-2 collagen, aggrecan, and SOX9 mRNAs was significantly greater in Group II than in the control group (p < 0.023).

**Conclusions:**

This study demonstrated that spontaneous hyaline cartilage regeneration can be induced *in vivo *in an osteochondral defect created in the femoral condyle by means of implanting the DN gel plug at the bottom of the defect so that an approximately 2-mm deep vacant space was intentionally left in the defect. This fact has prompted us to propose an innovative strategy without cell culture to repair osteochondral lesions in the femoral condyle.

## Background

Articular cartilage defects are a significant and increasing health care concern. It has been a commonly belief that hyaline cartilage tissue cannot spontaneously regenerate *in vivo *[1,2]. Therefore, the most progressive strategy to repair the articular cartilage defect is to fill an osteochondral defect with a tissue-engineered cartilage-like tissue or a cell-seeded scaffold material [3-6]. However, the cell culture procedures with the mammalian-derived materials/molecules include a possible risk of zoonosis transmission. In addition, it has been pointed out that this strategy has various realistic problems, including two-stage surgeries, a long period until weight bearing, an enormous amount of cost to establish a tissue-engineering industry system, possibly high medical fee for patients [7-10]. Under the similar strategy, some investigators have recently tried to fill up an osteochondral defect with acellular polymer scaffolds to induce cartilage cell regeneration inside it [11-14]. However, the results of these experimental trials are not favorable and are not indicated for clinical use. Thus, functional repair of articular osteochondral defects remains a major challenge not only in the field of knee surgery but also in tissue regeneration medicine.

We paid attention to the fact that sufficient fibrocartilage tissue can be regenerated in an osteochondral defect by creating many thin holes that penetrate the subchondral bone at the base of the defect in order to create bleeding from the bone marrow and subsequent clot formation ("Microfracture" technique). These induced mesenchymal stem cells have a high potential for cartilage regeneration [15]. In addition, recent studies have showed that, in autologous chondrocyte transplantation, quality of the tissue located just beneath the transplanted cells significantly affects quality of the regenerated cartilage [16,17]. In an *ex vivo *study, Engler et al [18] reported that elasticity of the material on which cultured cells attach directs stem cell differentiation: e.g., elastic materials induce differentiation to the cartilage tissue, and stiff materials induce differentiation to the bone tissue. Therefore, we hypothesize that a bioactive elastic material implanted in a chondral defect can stimulate and support hyaline cartilage regeneration.

We focused our research on an originally developed PAMPS/PDMAAm double-network (DN) hydrogel composed of poly-(2-Acrylamido-2-methylpropanesulfonic acid) (PAMPS) and poly-(N,N'-Dimetyl acrylamide) (PDMAAm) [19]. In our previous study validating the implant and its use in a large osteochondral defect created in the patellofemoral (PF) joint of the rabbit knee [20], we found that spontaneous hyaline cartilage regeneration occurred *in vivo *in the defect within 4 weeks after surgery when a PAMPS/PDMAAm DN gel plug was implanted at the bottom of the defect so that a 1.5 to 3.5-mm deep vacant space was intentionally left in the defect. In the clinical field, however, the joint that the most frequently requires a cartilage regeneration therapy is not the PF joint but the femorotibial (FT) joint. The PF and FT joints are anatomically, morphologically, and biomechanically different. Therefore, it is needed to clarify whether the spontaneous hyaline cartilage regeneration occurs in the FT joint. The purpose of this study is to clarify whether the spontaneous hyaline cartilage regeneration can be induced *in vivo *in a large osteochondral defect created in the medial femoral condyle of the FT joint by means of implanting a PAMPS/PDMAAm DN gel plug at the bottom of the defect.

## Methods

### 1) **Materials**

The PAMPS/PDMAAm DN hydrogel is a kind of interpenetrating network gel, but with an asymmetric structure: The first PAMPS network, which is rigid and brittle, is composed of densely cross-linked polyelectrolyte, and the second PDMAAm network, which is soft and ductile, consists of loosely or even non-crosslinked neutral polymers. The PAMPS/PDMAAm DN gel is strong enough to create an implantable plug, because the compressive fracture strength and the elastic modulus of the DN gel are 3.1 MPa and 0.2 MPa, respectively [21,22]. The material properties do not deteriorate in implantation into the subcutaneous tissue for 6 weeks [21]. The PAMPS network in this DN gel is negatively charged and has sulphonic acid bases, being similar to proteoglycans in normal cartilage. Our previous implantation test has shown that this DN gel is so bioactive that it induces cell infiltration in the muscle tissue at 1 week without any toxic effects for 6 weeks [23]. In addition, the PAMPS/PDMAAm DN gel surface can enhance differentiation of chondrogenic ATDC5 cells into chondrocytes in the *in vitro *condition [20,24].

The DN gel was synthesized by coauthors (T.K. and J.P.G) in Department of Biological Sciences, Hokkaido University Graduate School of Science, using the previously reported two-step sequential polymerization method [19]. After polymerization, the DN gel was immersed in pure water for 1 week and the water was changed 2 times every day to remove any un-reacted materials. From the DN gel, we created cylindrical plugs having a 2.7-mm diameter and an 8-mm length.

### 2) **Study design**

A total of 25 mature female New Zealand White rabbits, weighing 3.6 ± 0.4 kg, were used in this study. Animal experiments were carried out in the Institute of Animal Experimentation, Hokkaido University School of Medicine under the Rules and Regulation of the Animal Care and Use Committee, Hokkaido University School of Medicine.

**Figure 1:**
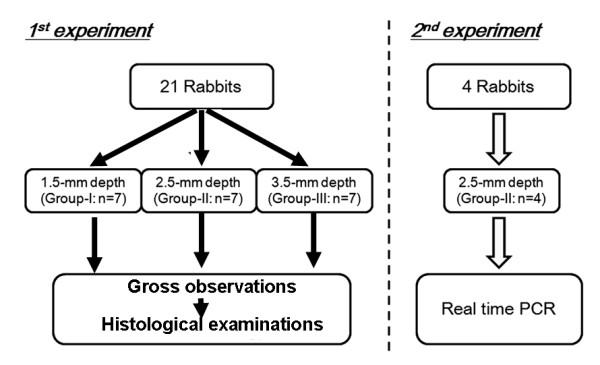
**Flowchart to explain the study design**.

**Figure 2:**
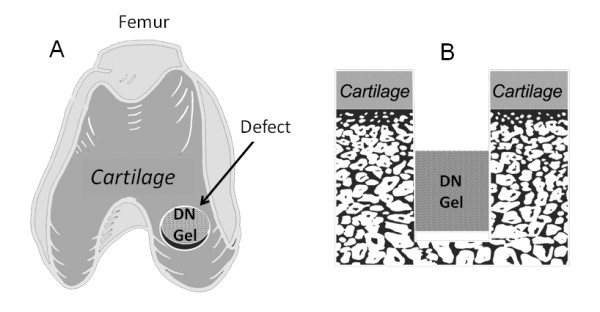
**How to induce cartilage regeneration**. **A**: We created a cylindrical osteochondral defect having a diameter of 2.4-mm in the medial condyle of the FT joint. Then, we implanted a double network (DN) gel plug into a bottom of the defect. **B**: A schematic cross-section of the osteochondral defect into which the plug was implanted. Note that a defect having a few millimeter depth from the cartilage surface remained after surgery.

This experimental report was composed of 2 studies (Figure 1). In the first study, we divided 21 out of the 25 rabbits into 3 groups (Groups I, II, and III) of 7 animals each in order to clarify the effect of plug position (depth from the articular surface) in the defect on quality of the regenerated cartilage. An operation for each animal was performed under intravenous anesthesia (pentobarbital, 25 mg/kg) and sterile conditions. In the bilateral knees of each animal, we created a cylindrical osteochondral defect having a diameter of 2.4-mm at the center of the medial condyle of the FT joint, using a drill (Figure 2A). This diameter value was chosen as the maximal defect diameter that we could create on the cartilage surface without intra- or post-operative fracture of the medial condyle, because the width of the medial condyle around the defect was approximately 4.5 mm. Then, we implanted the PAMPS/PDMAAm DN gel plug into a defect in the right knee so that a vacant space of 1.5-mm depth (in Group I), 2.5-mm depth (in Group II), or 3.5-mm depth (in Group III) was left (Figure 2B). The actual depth was precisely measured in the histological sections after sacrifice. According to the measured results, "the mean ± the standard deviation" of the real depth were 1.49 ± 0.28 mm in Group I, 2.44 ± 0.27 mm in Group II, and 3.46 ± 0.31 mm in Group III. The cartilage thickness was approximately 0.5 mm around the defect. In the left knee, we created the defect having the same depth as the right knee, and we did not apply any treatment to obtain the non-treated control (Control group). The incised joint capsule and the skin wound were closed in layers with 3-0 nylon sutures, and an antiseptic spray dressing was applied. Postoperatively, each animal was allowed unrestricted activity in a cage (310 × 550 × 320 mm) without any joint immobilization. In each group, all rabbits were sacrificed by pentobarbital injection at the 4-week period, and we performed the gross and histological evaluations using the grading scale reported by Wayne et al [14] as well as immunohistochemical observations for the bilateral knees.

The second study was conducted using 4 rabbits, based on the results of the first study. The aim of the second study using real-time PCR analysis was to confirm gene expression of type-2 collagen, aggrecan, and SOX9 in the tissue regenerated in the defect of Group II in comparison with the non-treated control knees, because the degree of spontaneous cartilage regeneration was the greatest in Group II among the tested groups in the first study. The same surgical treatments as performed in Group II of the first study were carried out in the bilateral knees, respectively. Immediately after sacrifice at 4 weeks, total RNA was extracted from the tissues regenerated in the defect created in the bilateral knees.

### 3) Statistical Analysis

The scores for each specimen were assessed for statistical differences using one-way analysis of variance with the Fisher's protected least significance difference for post hoc multiple comparisons. A commercially available software program (StatView, SAS Institute, NC) was used for statistical calculation. The significance level was set at p = 0.05.

### 4) Examination methods

#### Gross observation for in vivo regenerated tissues

Immediately after sacrifice, the tissue regenerated in the osteochondral defect was quantitatively evaluated with the grading scale reported by Wayne et al [14]. Gross appearance of each defect on the femoral condyle was graded for coverage (4 points), tissue color (4 points), defect margins (4 points), and surface (4 points). Thus, the maximum total score was 16 points.

#### Histological and immunohistochemical examinations

A distal portion of the resected femur was fixed in a 10% neutral buffered formalin solution for 3 days, decalcified with 50 mM EDTA for a period of 3-4 weeks, and then cast in a paraffin block. The femur was sectioned perpendicular to the longitudinal axis, and stained with hematoxylin-eosin and Safranin-O. For immunohistochemical evaluations, monoclonal antibody (anti-hCL(II), purified IgG, Fuji Chemical Industries Ltd, Toyama, Japan) was used as primary antibodies. Immunostaining was carried out according to the manufacturer's instructions using the Envision immunostaining system (DAKO Japan, Kyoto, Japan). Finally, the sections were counterstained with hematoxylin. Histology was evaluated with the scoring system reported by Wayne et al [14], which was composed of matrix points (4 points), cell distribution points (3 points), smoothness points of the surface (4 points), safranin O stain points (4 points), safranin O-stained area points (4 points). Thus, the maximum total score was 19 points.

#### Real time polymerase chain reaction (PCR) analysis

Total RNA was extracted from the tissues regenerated in the defect, using the RNeasy mini kit (Qiagen Inc., Valencia, CA). RNA quality from each sample was assured by the A260/280 absorbance ratio. The RNA (100 ng) was reverse-transcribed into single strand cDNA using PrimeScript^® ^RT reagent Kit (TakaraBio, Ohtsu, Japan). The RT reaction was carried out for 15 minutes at 37 degrees Celsius and then for 5 seconds at 85 degrees Celsius. All oligonucleotide primer sets were designed based upon the published mRNA sequence. The expected amplicon lengths ranged from 93 to 189 bp. The sequences of primers used in real time PCR analyses for rabbit regenerative tissues were as follows: type-2 collagen forward GACCATCAATGGCGGCTTC; reverse CACGCTGTTCTTGCAGTGGTAG. Aggrecan forward GCTACGACGCCATCTGCTAC; reverse GTCTGGACCGTGATGTCCTC. SOX9 forward AACGCCGAGCTCAGCAAGA; reverse TGGTACTTGTAGTCCGGGTGGTC. GAPDH forward CCCTCAATGACCACTTTGTGAA; reverse AGGCCATGTGGACCATGAG. The real time PCR was performed in Thermal Cycler Dice^® ^TP800 (TakaraBio, Ohtsu, Japan) by using SYBR^® ^Premix Ex TaqTM (TakaraBio, Ohtsu, Japan). cDNA template (5 ng) was used for real time PCR in a final volume of 25 microlitter. cDNA was amplified according to the following condition: 95 degrees Celsius for 5 sec and 60 degrees Celsius for 30 sec at 40 amplification cycles. Fluorescence changes were monitored with SYBR Green after every cycle. A dissociation curve analysis was performed (0.5 degrees Celsius/sec increase from 60 to 95 degrees Celsius with continuous fluorescence readings) at the end of cycles to ensure that single PCR products were obtained. The amplicon size and reaction specificity were confirmed by 2.5% agarose gel electrophoresis. The results were evaluated using the Thermal Cycler Dice^® ^Real Time System software program (TakaraBio, Ohtsu, Japan). Glyceroaldehyde-3-phosphate dehydrogenase (GAPDH) primers were used to normalize samples.

## Results

### Gross observation of the joint surface repair

**Figure 3:**
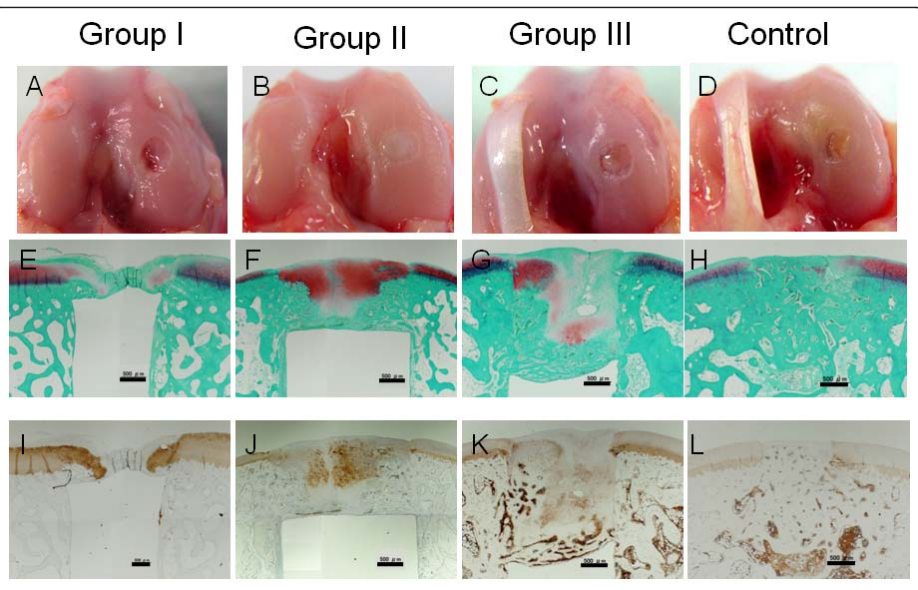
**Gross observations of the joint surface (A-D), histological findings with Safranin-O staining (E-H), and immunohistological findings with type-2 collagen staining (I-L)**. Black scale bars show a length of 500 micrometers. In Groups II, the defect was filled with the hyaline cartilage tissue rich in proteoglycan and type-2 collagen. In Groups I and III, we observed the cartilage tissue in the defect although it was not homogenous in Safranin-O staining or type-2 collagen staining.

In gross observations, the knee joint did not show any inflammation or any pathological changes. The defect was filled with a white opaque tissue in Groups II, while the defect was insufficiently filled with white or reddish, opaque, patchy tissues in Groups I and III, (Figure 3A-C). The untreated defect in Control group showed white or reddish, opaque, patchy, stiff tissues, independent of the depth (Figure 3D).

### Histological and immunohistological evaluations

Low magnification histology (Figure 3E-L) showed that the untreated (control) defect was filled with the fibrous and bone tissues, while a small amount of the proteoglycan-rich tissue was occasionally and irregularly seen in these tissues (Figure 3H). The type-2 collagen expression was not found in the tissue regenerated in the untreated defect, except for a limited amount in the peripheral portion (Figure 3L). On the other hand, the defect of Group II was filled by a sufficient volume of the proteoglycan-rich tissue with regenerated subchondral bone tissue (Figure 3F). The detailed surface histomorphological change of each specimen was described in the Table 1. The immunohistochemical observation showed that the type-2 collagen was abundantly expressed in the proteoglycan-rich tissue (Figure 3J). These findings showed that the hyaline cartilage tissue was regenerated in the defect of Group II. Regarding the defect of Groups I and III, we observed the cartilage tissue in the defect although the tissue was not homogenous in Safranin-O staining or type-2 collagen staining.

**Table 1:** Surface evaluations on the histological sections of DN gel-implanted specimens in each group.

	Group I	Group II	Group III
Smooth level/with normal	1	4	4
(normal surface)			
Smooth but raised	0	1	2
(overgrowth surface)		(0.155)	(0.366 - 0.380)
Irregular	6	2	1
(cleft/defect remained)	(0.204 - 0.405)	(0.121 - 0.146)	(0.481)

The range of the maximum depth or overgrowth in each specimen was noted in the parentheses.

**Figure 4:**
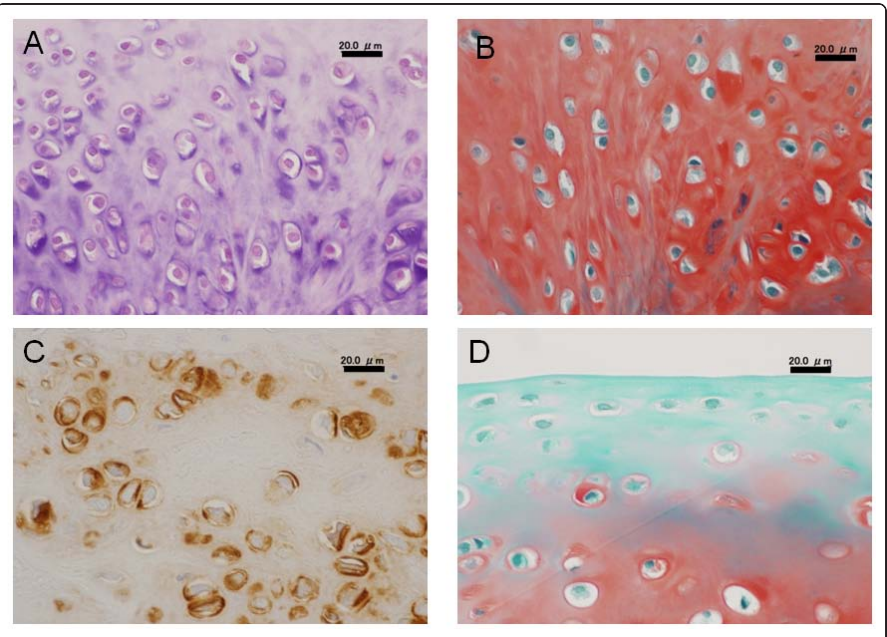
**High magnification histology of Group II**. Black scale bars show a length of 20.0 micrometers. Large round cells rich in cytoplasm were scattered singly or as an isogenous group in a proteoglycan-rich matrix (A and B). In these cells, type 2 collagen was richly expressed (C). At the superficial layer, cells were relatively small and aligned parallel to the surface (D). The most superficial layer was devoid of cells, resembling the lamina splendens in the normal articular cartilage (D).

In high magnification histology of Group II, fairly large round cells rich in cytoplasm were scattered singly or as an isogenous group in a proteoglycan-rich matrix (Figure 4A and 4B). In these cells, type-2 collagen was richly expressed (Figure 4C). At the superficial layer in this tissue, cells were relatively small and sparse, while some cells were aligned as cell columns parallel to the surface (Figure 4D). In addition, the most superficial layer was devoid of cells, resembling the lamina splendens in the normal articular cartilage (Figure 4D).

### Quantitative evaluations of gross appearance and histology

**Figure 5:**
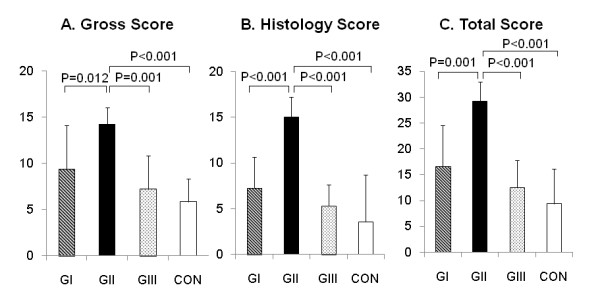
**Quantitative evaluations of gross appearance and histology**. Concerning each score, Group II was significantly greater than Groups I, III, and Control. While there were no significant differences among Groups I, III, and Control.

Concerning the gross appearance score, Group II was significantly greater than Groups I, III, and Control (p = 0.0119, p = 0.0006, and p < 0.0001, respectively) (Figure 5A). Regarding the histology score, Group II was significantly greater than the other groups (p = 0.0004, p < 0.0001, and p < 0.0001, respectively) (Figure 5B). Thus, the total score showed that Group II was significantly greater than the other groups (p = 0.0007, p < 0.0001, and p < 0.0001, respectively) (Figure 5C). In each score, there were no significant differences among Groups I, III, and Control.

### Real time PCR analysis

**Figure 6:**
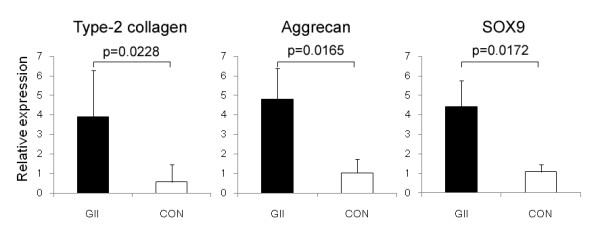
**Real time PCR analysis**. The degree of expression of type-2 collagen, Aggrican, and SOX9 mRNAs was significantly greater in the regenerated tissue of Group II than that of Control Group.

In the real time PCR analysis performed at 4 weeks, the degree of expression of type-2 collagen, aggrecan, and SOX9 mRNAs was significantly greater in the regenerated tissue of Group II than that of Control Group (p = 0.0228, p = 0.0165, and p = 0.0172, respectively) (Figure 6). The expressions were seldom seen in the tissues regenerated in the untreated defect.

## Discussion

The present study demonstrated, first, that spontaneous hyaline cartilage regeneration can be induced *in vivo *in a large osteochondral defect created even in the FT joint by means of implanting a cylindrical PAMPS/PDMAAm DN gel plug at the bottom of the defect in the rabbit so that an approximately 2-mm deep vacant space was intentionally left in the defect. This fact suggested that the spontaneous hyaline cartilage regeneration using the DN gel implantation is not a specific phenomenon in the PF joint but common one in the diarthrodial joints. Secondly, this study showed that the regeneration effect was affected by the position (depth) of the implanted gel plug in the defect. We have reported that mechanical environment generated by repetitive compression forces during weight bearing and the elastic properties of the DN gel located at the bottom of the defect is a significant factor that differentiates stem cells that exist in the defect to chondrocytes [25]. Therefore, it is considered that the position (depth) of the implanted gel plug in the defect is one of significant factors that affect the mechanical environment in the defect, resulting in the outcome of the hyaline cartilage regeneration. In addition, the second study showed that the gene expression measured in Group II correlated to the better results in spontaneous cartilage regeneration. This result suggested that the treatment with a DN gel induced spontaneous hyaline cartilage regeneration by applying unknown effects in the gene level on stem cells infiltrating in the defect.

We have speculated the reasons why the regeneration effect was affected by the position (depth) of the implanted gel plug in the defect. First, our previous study has demonstrated that the cartilage regeneration occurs in the blood clot, which is containing mesenchymal stem cells and various cytokines [20]. We believe that a sufficient amount of blood clot must be formed in the defect immediately after surgery for spontaneous cartilage regeneration. Therefore, the reason why the cartilage regeneration was not induced in Group I of the present study (a vacant space of 1.5-mm depth) is considered that the space was so narrow that blood clot could not be sufficiently formed in the vacant space. Secondly, we consider that the reason why the cartilage regeneration was not induced in Group III (3.5-mm depth) of the present study but in Group II (2.5-mm depth) can be explained by the difference of biomechanical conditions. Kelly et al. reported that mechanical signals play an important role in differentiation of bone-marrow derived stem cells [26]. Engler et al. reported that matrix elasticity influences the differentiation of mesenchymal stem cells [18]. In addition, appropriate repetitive compressive stress significantly enhances chondrocyte proliferation as well as aggrecan and collagen synthesis in chondrocytes [27-30]. Recently, we have found that joint motion is needed to induce the spontaneous hyaline cartilage regeneration in the osteochondral defect using the DN gel [25]. The joint motion generates repetitive compression forces to the tissue regenerated in the defect. The repetitive forces create mechanical environment in the regenerated tissue, being affected by the location (the depth) and the stiffness of the gel plug that located beneath the tissue. Thus, we speculate that the mechanical microenvironment in the tissue of Group II was appropriate for cartilage regeneration, but that the mechanical microenvironment in the defect of Group III was inappropriate.

The results of the present study have prompted us to propose an innovative strategy to clinically repair various osteochondral lesions in the femoral condyle with the DN gel implantation and induction of the spontaneous cartilage regeneration. We should note that this therapeutic strategy is new and completely different in the concept from the current progressive strategies that completely fill the defected space with the tissue-engineered cartilage tissue, cell-seeded scaffold material implantation, or acellular polymer scaffolds with signaling molecules [11-13]. Numerous problems exist for current treatment strategies for chondral and osteochondral defects including but not limited to donor site morbidity, multiple surgeries required, prolonged limitations in activity, and significant financial costs [7-10]. We believe that the spontaneous regeneration strategy has potential to solve almost all of the above-described problems of the current progressive strategies. Therefore, the spontaneous regeneration strategy should be studied as a realistic research focus in greater detail in the near future. For example, we reported that the depth of 2.5 mm is optimal for lesions in the rabbit medial femoral condyle in this study. However, we did not analyze the relationship among the depth of the plug, the depth of the whole defect space, and the height of the plug. In addition, we did not analyze on the influence of the ratio of cartilage thickness to depth of lesion on cartilage regeneration. For the possible clinical use of this treatment strategy, further studies should be conducted to clarify these issues in the near future.

Regarding the safety of the PAMPS/PDMAAm DN gel as a biomaterial, we conducted a pellet implantation test into the para-vertebral muscle [23], according to the guideline for biological evaluation of the safety of biomaterials, which had been published by the Ministry of Health, Labour and Welfare, Japan. Although this DN gel implantation induced a mild cell infiltration at 1 week, the degree of the inflammation significantly decreased into the same degree as that of the negative control at 4 and 6 weeks. We also cultured ATDC5 cells on the PAMPS/PDMAAm DN gel [20,24]. No harmful effects due to the DN gel surface were detected. We believe that the PAMPS/PDMAAm DN gel is a safe biomaterial. However, we have not completed to establish the clinical safety of this DN gel as an implant. Further studies are needed to establish the clinical safety of this gel in the near future.

There are some limitations in this study. The first limitation is that the number of animals was insufficient in the second study because the statistical power was 0.55, although the power in the first study was sufficient (0.9). However, we should note that the second study showed the statistical significant difference beyond the low power. Therefore, the number of 4 rabbits was acceptable as an experimental study. The second limitation is that we did not perform long-term observation of the regenerated cartilage above the DN gel or a border between the cartilage and the original tissue. A long-term evaluation study is needed to be performed immediately after this study. The third limitation is that we have not completely clarified the mechanism of the spontaneous cartilage tissue regeneration by the DN gel implantation. Further studies are needed in the near future in order to clarify the *in vivo *comprehensive mechanism of the spontaneous hyaline cartilage regeneration induced in a large osteochondral defect by means of implanting a cylindrical PAMPS/PDMAAm DN gel plug at the bottom of the defect.

## Conclusions

This study demonstrated that spontaneous hyaline cartilage regeneration can be induced *in vivo *in an osteochondral defect created in the femoral condyle by means of implanting the DN gel plug at the bottom of the defect so that an approximately 2-mm deep vacant space was intentionally left in the defect. This fact has prompted us to propose an innovative strategy without cell culture to repair osteochondral lesions in the femoral condyle.

## Financial competing interests

The authors declare that they have no competing interests.

## Authors' contributions

MY performed animal experiments. KY designed the study, participated in the study, and drafted the manuscript. NK and KA participated in designing the study and instructed animal experiments. SO performed the PCR analysis. TK and JPG created the DN-gel material. All authors read and approved the final manuscript.

## Pre-publication history

The pre-publication history for this paper can be accessed here:

http://www.biomedcentral.com/1471-2474/12/49/prepub
